# Development of MOF Based Recyclable Photocatalyst for the Removal of Different Organic Dye Pollutants

**DOI:** 10.3390/nano13020336

**Published:** 2023-01-13

**Authors:** Narasimharao Kitchamsetti, Chidurala Shilpa Chakra, Ana Lucia Ferreira De Barros, Daewon Kim

**Affiliations:** 1 Department of Electronic Engineering, Institute for Wearable Convergence Electronics, Kyung Hee University, 1732 Deogyeong-daero, Giheung-gu, Yongin 17104, Republic of Korea; 2 Center for Nano Science and Technology, Institute of Science and Technology, JNTU Hyderabad, Hyderabad 500090, India; 3 Laboratory of Experimental and Applied Physics, Centro Federal de Educação Tecnológica Celso Suckow da Fonseca, Av. Maracanã Campus 229, Rio de Janeiro 20271-110, Brazil

**Keywords:** solvothermal, metal organic framework, photocatalyst, nanosheets, organic pollutant

## Abstract

The preparation of metal organic frameworks (MOFs) has come to the forefront in recent years because of their outstanding physical and chemical properties. Many MOFs such as Zn, Co, Ni, Fe, and Ag, etc., have been successfully synthesized. In this work, we followed the solvothermal assisted route to synthesize Ag-MOF (abbreviated as AMOF) nanosheets and then applied them as a photocatalyst to remove different organic pollutants, namely methyl orange (MO), crystal violet (CV), and methylene blue (MB). Chemical composition, optical properties, morphology, and microstructural analysis were analyzed using XPS, UV-visible spectrophotometer, FESEM, TEM, and EDS, respectively. The structural properties of AMOF nanosheets were studied by X-ray diffraction (XRD). Nitrogen adsorption and desorption isotherm analysis were utilized to evaluate the specific surface area and pore size of the AMOF nanosheets. Further, AMOF nanosheets showed notable photocatalytic performance for various dye pollutants degradation. The results confirmed 74.5, 85.5, and 90.7% of MO, CV, and MB dye pollutants removal after 120 min of irradiation with the rate constants (k) of 0.0123, 0.0153, and 0.0158 min^−1^, respectively. The effect of superoxide radicals (O_2_^−^) and photogenerated holes (h^+^) on the organic dye pollutants removal was investigated using radical scavenger trapping studies. Moreover, the stability study also confirmed the recyclability of the photocatalyst. Therefore, the findings of this research present a realizable method to grow AMOF photocatalyst for successful degradation of various dye pollutants.

## 1. Introduction

Water is one of the most important essential requirements for every living organism in the ecosystem. Over recent years, the environment has been contaminated severely by organic pollutants found in industrial effluents, including various cationic and anionic dyes [1,2]. With the current fast-industrialization and technological development, there is an urgent need to protect the environment from pollution especially water pollution [3]. Industries all over the planet produce wastewater in huge amounts which contains wastes mainly of organic and inorganic mixtures. The inadequate treatment of all of these wastes poses a serious threat to human health and the quality of water bodies [4,5]. Particularly, organic pollutants are very difficult to degrade, and extremely toxic [6,7]. Thus, to degrade organic pollutants in wastewater efficiently, adsorption, coagulation/flocculation, chemical oxidation, ion exchange, and photocatalysis methods have been mainly employed [8,9]. Among all the methods, photocatalysis has been determined as an effective technique to remove the contaminants from wastewater due to the generation of radical scavengers such as O_2_^−^, and OH^−^ in large amounts which assist in the conversion of dye pollutants into H_2_O and CO_2_ [10,11]. Until now, various forms of metal oxides and composites have been utilized as photocatalysts for the removal of various organic dye contaminants [12]. However, this type of catalyst is subjected to recombination of electron and hole pairs, easy clustering, and low energy consumption [13]. Therefore, with the widespread awareness on human health and environmental protection, green technologies to remove pollutants from wastewater at low cost and high efficacy are in huge demand [14]. Especially, there is an urgent requirement to develop a photocatalyst with high performance to handle these pollutants [15].

Metal–organic frameworks (MOFs) constructed with a metal ions and organic linkers have been shown to be a potential material for various applications such as sensing, energy storage, catalysis, proton conduction, and gas storage [16,17,18,19]. Furthermore, some MOFs illustrate a semiconducting mechanism in the presence of light, indicating that they may be probably utilized as photocatalysts [20,21]. Compared to traditional inorganic semiconductors, MOFs have various merits that include catalytically active organic linkers and unsaturated metal ion sites [22]. The recombination of an electron and hole pair is due to the termination of the structural deficiencies of MOFs [23]. First, Alvaro et al. and his group studied phenol degradation using MOF-5 as a photocatalyst [24]. Later Liu and Xia et al. demonstrated the removal of organic dyes using an MOF as a photocatalyst [25,26]. Further, Zhao et al. reported a preparation of a heterogeneous photocatalyst for organic dye pollutants removal [27]. Lately, Dong et al., demonstrated the removal of organic dyes using 2D Zn-MOF as a prominent photocatalyst [10]. However, these reports have initiated thought on the great potential of MOFs in the photocatalytic water treatment world. Even though reports, although very limited, are available on MOFs as photocatalysts, there remains a huge potential to explore the different types of MOFs to degrade various organic pollutants in the near future. For instance, the available reports on MOF-based photocatalysts demonstrated superior photocatalytic performance in removing various organic pollutants using H_2_O_2_ as a cocatalyst. Nevertheless, using such cocatalysts in a large amount to degrade the present organic dyes wastewater is not an environmental-friendly approach. Therefore, there is an urgent need to design a photocatalysts that can deliver high photocatalytic activity without the use of any other cocatalyst or photosensitizer [27].

Ni, Zn, Co, Cu, Ag, and Fe based MOF materials have been well studied in the energy and environmental fields. All these MOFs can be synthesized by different chemical routes like sol-gel, hydrothermal, and solvothermal techniques [28,29,30,31,32]. Out of all of these conducted MOFs, Ag-MOF (abbreviated as AMOF) could be the potential attractive candidate in multifunctional applications because of high electrical conductivity, active surface sites, low resistance, and its bonding nature [33]. Due to its extraordinary properties, AMOF has been utilized in different applications such as biosensing [34], photocatalysts [35], battery studies [36], and filtration membranes for SERS detection [37].

Very little literature is available on the chemical preparation of Ag based nanostructures and their utilization in multifunctional fields [6,33,34,35,36] and detailed study on photocatalytic removal of MB, MO, and CV dye contaminants under Xenon light illumination has not yet been reported. According to our knowledge and belief, the preparation of AMOF, its characterization, and its application as a photocatalyst to remove different organic dye contaminants is reported here for the first time. In this study, porous AMOF nanosheets were prepared via the solvothermal method under reasonable conditions. The optical and structural properties of AMOF nanosheets were investigated using UV-visible spectroscopy, high resolution transmission electron microscopy, field emission scanning electron microscopy, X-ray photoelectron spectroscopy, X-ray diffraction, and nitrogen adsorption and desorption isotherm measurements. Photocatalysis of AMOF nanosheets was estimated by the degradation of crystal violet (CV), methylene blue (MB), and methyl orange (MO) pollutants in the presence of visible light illumination. Dye removal percentages of 74.5, 85.5, and 90.7% for MO, CV, and MB dyes were obtained from the optimized photocatalytic activity. The AMOF nanosheets revealed a high percentage of dye removal in the case of MB compared to the other dyes. Furthermore, the stable photocatalytic activity and recyclability of AMOF nanosheets were analyzed using XRD and FTIR spectra without the creation of other organic pollutants and structural changes. The big influence of superoxide radicals and photogenerated holes on the photocatalytic removal of dye pollutants were studied using radical scavenger trapping experiments. Overall, our study may open a new route to investigate MOF based materials as favorable photocatalysts for the development of a low cost and environmentally friendly approach for the removal of organic contaminants.

## 2. Experimental Procedure

### 2.1. Chemicals and Reagents

All the chemicals were of analytical grade and used as is without further purification. Methanol, *N*,*N*-dimethylformamide (DMF, ≥99%), terephthalic acid (H_2_BDC, ≥99%), and silver nitrate (AgNO_3_, ≥99%) were procured from Sigma-Aldrich (Seoul, South Korea).

### 2.2. Synthesis of Ag-MOF

**Figure 1:**
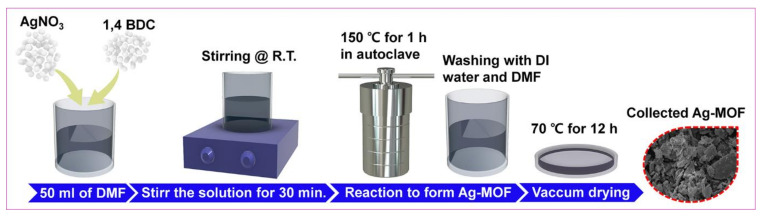
Schematic demonstration of AMOF formation.

In brief, AgNO_3_ solution of 0.2 M was mixed with DMF (50 mL) and kept under vigorous stirring. An amount of 0.2 M of H_2_BDC was added to the above solution and stirred further for half an hour. Then, the whole solution was moved to an autoclave. After heating for 1 h at 150 °C, the product was allowed to cool to room temperature. Further, the resultant (i.e., Ag-MOF) was washed via centrifugation using DMF and DI water several times thereby obtaining the desired product after drying under vacuum at 70 °C. Moreover, the in detailed design of Ag-MOF is explained in Figure 1.

### 2.3. Characterization

X-ray diffraction (XRD, Cu-Kα radiation, M18XHF-SRA, MAC Science, Yokohama, Japan) was used to check the nature of the samples and their crystal structure. The electronic nature and chemical composition of AMOF were investigated by an X-ray photoelectron spectrometer (XPS, Thermo Electron Multilab 2000, East Grinstead, UK) with microfocus monochromated Al Ka X-rays. The surface morphological analyses were carried out using a high-resolution transmission electron microscope (HRTEM, JEM 2100F, Oxford Instruments INCA, High Wycombe, UK) and a field emission scanning electron microscope (FESEM, Carl Zeiss, LEO SUPRA 55, Oberkochen, Germany). The pore size and surface area of AMOF were examined by N_2_ adsorption–desorption isotherm (BET) and Barrett–Joyner–Halenda (BJH) approaches in an automated gas sorption analyzer (BELSORP-max (MP)). The presence of functional groups in the sample was determined by a Fourier transform infrared (FTIR) spectrometer (Perkin-Elmer, Spectrum One System, Hudson, NY, USA). The optical properties were explored using a UV-visible spectrophotometer (Shimadzu UV-2600, Seoul, South Korea). Initially, the AMOF photocatalyst and dye concentrations were adjusted using the crystal violet (CV) dye. Further, these optimized concentrations of photocatalyst and dye were utilized to explore the removal of methylene blue (MB) and methyl orange (MO) in the presence of 300 W Xe lamp (Newport, λ ≥ 420 nm) illumination. Initially the concentration of dye was optimized by varying the CV dye concentration from 10 to 30 ppm. Similarly, the mass of photocatalyst was also optimized by varying the concentration from 10 to 50 mg using 10 ppm concentration of CV dye. To achieve an adsorption–desorption equilibrium between the photocatalyst and dye pollutant, the solution was stirred continuously for 1 h [38], and further the solution was exposed to 300 W Xenon lamp irradiation. The samples were collected after irradiation at regular intervals and the UV-visible absorption spectra analyzed of all the collected samples. The initial concentration (C_0_) of dyes (i.e., MO, CV, and MB) solution and the concentration at reaction time t (C_t_) were evaluated to measure the percentage of dye removal using the below equation [39].

(1) $$\mathrm{Dye}\;\mathrm{degradation}\;\left(\%\right)=\frac{C_{0}-C_{t}}{C_{0}}\times 100$$

## 3. Results and Discussion

**Figure 2:**
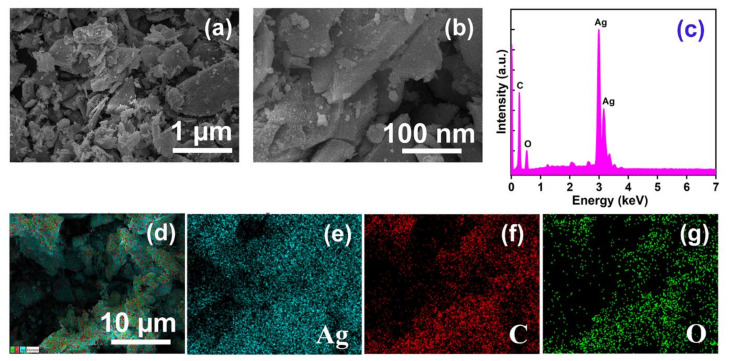
FESEM analysis of AMOF at (**a**) low, and (**b**) high magnification. (**c**) EDS spectra of AMOF, and (**d**–**g**) elemental mapping of Ag, C, and O, respectively in AMOF nanosheets.

The morphology of AMOF exhibits a significant role in photocatalytic activity. The collection of non-uniform distributed particles initiates the sheet like morphology of AMOF, revealed in Figure 2a,b. The high resolution FESEM image (i.e., Figure 2b) proves that the AMOF nanosheet surface appears very rough according to its nature, providing some pores consisting of numerous nanoparticles. An effective surface area was delivered by the AMOF nanosheets for the photocatalytic mechanism. Further, the EDS spectrum of the AMOF nanosheet is shown in Figure 2c. The elemental mapping demonstrated in Figure 2d–g validates the existence of Ag, C, and O elements in the AMOF nanosheet. Further, no extra peaks were detected, which confirms the correct formation of the AMOF nanosheet.

**Figure 3:**
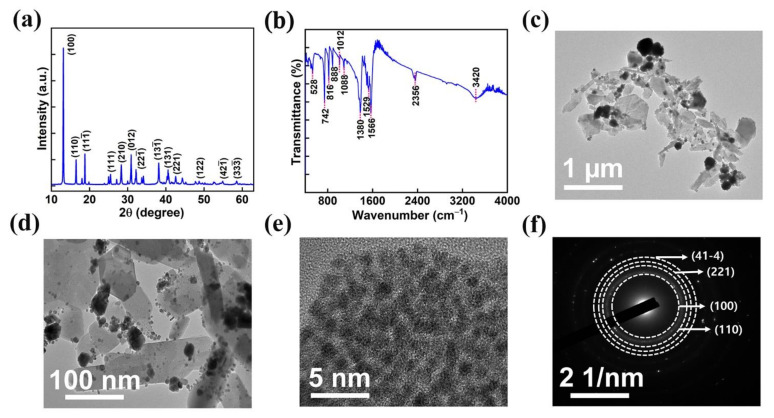
(**a**) XRD spectra, (**b**) FTIR analysis of AMOF, (**c**,**d**) TEM images at low and high magnifications. (**e**) HRTEM image and (**f**) SAED pattern of AMOF.

The phase purity and crystallinity of the AMOF nanosheet were investigated by utilizing XRD analysis. The diffraction pattern of AMOF is represented in Figure 3a. The peaks located at 2θ of 13.12, 16.52, 18.87, 25.48, 28.51, 31.05, 32.25, 34.04, 38.14, 40.95, 42.82, 54.86, and 58.66 correspond to the (100), (110), ($11\bar{1}$), (111), (210), (012), (22$\bar{1}$), (13$\bar{1}$), (131), (221), (122), (42$\bar{1}$), and (33$\bar{3}$) planes, respectively. The obtained results are in good agreement with CCDC 198096, further it confirms the successful construction of a monoclinic structure with the P21/c space group [40]. The FTIR spectra, shown in Figure 3b, validate the functional groups present in the AMOF nanosheets. The presence of water is confirmed by the broad vibrational −OH band observed at 3420 cm^−1^. The asymmetric and symmetric vibrations of the C=O carboxylate group represent the −COOH of terephthalic acid coordinated to a central atom through the bidenticity mode indicated by the intense absorption peaks at 1566 and 1529 cm^−1^. The absorption band recorded at 1380 cm^−1^ matches the C=C of the benzene ring. The bending vibrations of M−OH are indicated by the bands observed at 1088 and 1012 cm^−1^. Similarly, the band at 816 cm^−1^ resembles the carbon hydrogen bonds in benzene-1,4-dicarboxylic acid (BDC). Further, the presence of Ag–O is indicated by the existence of a band at 742 cm^−1^ [41].

Further, high-resolution transmission electron microscopy was used to analyze the morphology of the AMOF nanosheets. Figure 3c–e displays the TEM images of AMOF at diverse magnifications. TEM analysis confirms that the nonuniform particles are well amassed and integrated to exhibit a morphology of sheet-like structure. The SAED pattern with consecutive rings shown in Figure 3f reveals that AMOF is of polycrystalline nature and proves the formation of a monoclinic structure. Moreover, the results observed in TEM are well matched with the XRD analysis.

**Figure 4:**
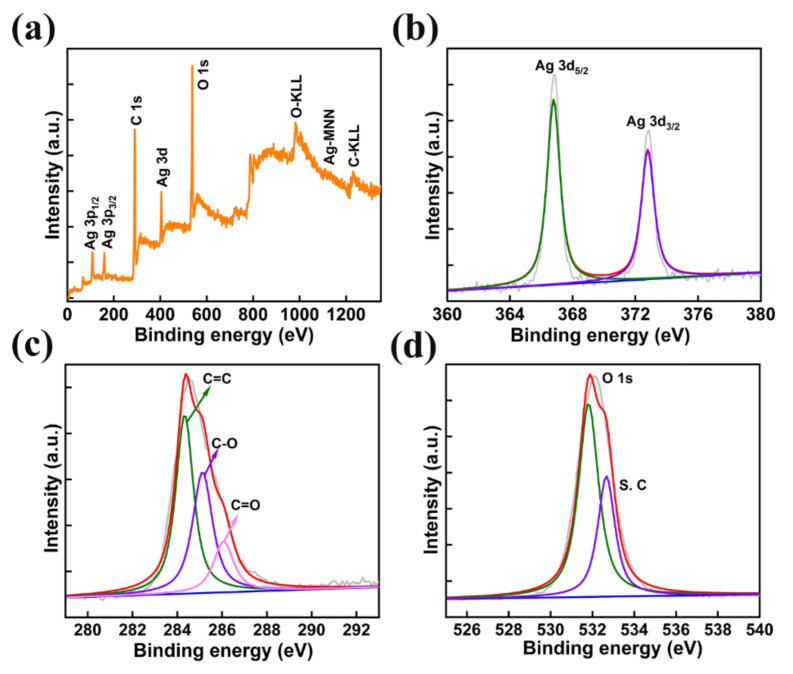
(**a**) XPS survey spectra of AMOF, (**b**) Ag 3d, (**c**) C 1s, and (**d**) O 1s spectra.

The chemical composition and electronic states of the prepared AMOF were investigated using X-ray photoelectron spectroscopy. The survey scan of AMOF shown in Figure 4a confirms the existence of Ag, C, and O elements. Further, the survey scan is well matched with the EDS analysis and elemental mapping. The peaks at binding energies of 366.78 eV (Ag 3d_5/2_) and 372.78 eV (Ag 3d_3/2_) specify the presence of Ag^+^ shown in Figure 4b. The energy separation between these two peaks is 6 eV, which depicts the existence of Ag in the AMOF nanosheets [42,43]. The C (1s) spectrum shown in Figure 4c reveals three deconvoluted peaks located at 284.28, 285.18, and 286.08 eV binding energy corresponding to C=C, C–O, C=O, respectively. Further, the O (1s) spectrum plotted in Figure 4d represents the two peaks at 531.88 and 532.68 eV belonging to the metal oxygen bond (Ag–O) and O–H groups, respectively [44].

**Figure 5:**
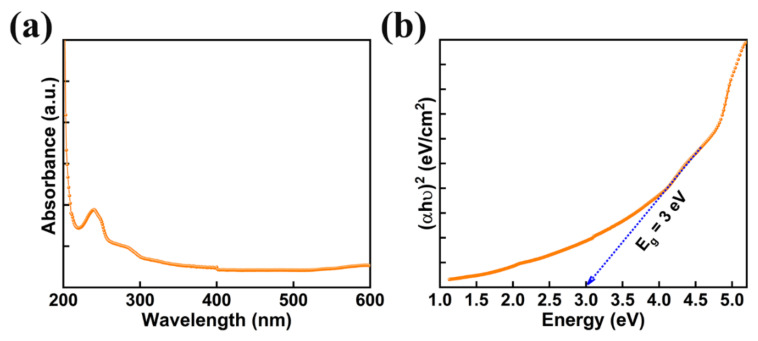
(**a**) UV-vis absorption spectra of AMOF nanosheets, and (**b**) bandgap measurements attained from the Tauc plot.

Photocatalysts with reasonable band gap and good absorption of light are beneficial for achieving exceptional photocatalytic performance. The absorption spectra of AMOF nanosheets was studied with UV-visible spectroscopy shown in Figure 5a. AMOF nanosheets display significant absorbance in the wavelength range of 250–800 nm. The absorption of light may occur due to numerous light scattering within the porous structure, which advances the chances of light utilization. Tauc’s plot can be drawn to estimate the band gap using the below formula [45].

(αhυ)^2^ = A (hυ − E_g_) (2)

where h is Planck’s constant, A is a constant, α is the coefficient of absorption, E_g_ is the bandgap, υ is the frequency of light, and hυ is the energy of the photons of the AMOF nanosheets. A bandgap of 3 eV was estimated from the (hυ) versus (αhυ)^2^ plot demonstrated in Figure 5b. Consequently, the reasonable bandgap and stronger light absorption of AMOF nanosheets may support using the energy of visible light more effectively.

**Figure 6:**
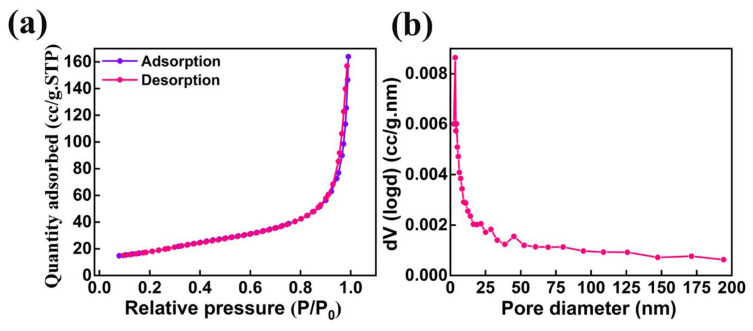
Surface area and pore size analysis of AMOF: (**a**) nitrogen adsorption–desorption isotherm and (**b**) pore size distribution.

The specific surface area and pore size distribution are the two major surface properties which play a significant role in the performance of photocatalytic activity. The nitrogen adsorption–desorption isotherm studies were analyzed to understand the formation of the porous nature of AMOF nanosheets. The AMOF nanosheets exhibits an average pore volume and Brunauer–Emmett–Teller (BET) surface area of 0.25 cm^3^/g and 62.7 m^2^/g, respectively. The analysis of the nitrogen adsorption–desorption isotherm reveals that the AMOF nanosheets follow a type IV isotherm with an extensive hysteresis loop experienced in the 0–1 pressure (P/P_0_) range, demonstrated in Figure 6a, which proves the formation of the mesoporous AMOF nanosheets. Figure 6b reveals the pore size distribution plot obtained from the isotherm, demonstrating that the pore diameter lies within the range of 3–4 nm for the majority of pores.

**Figure 7:**
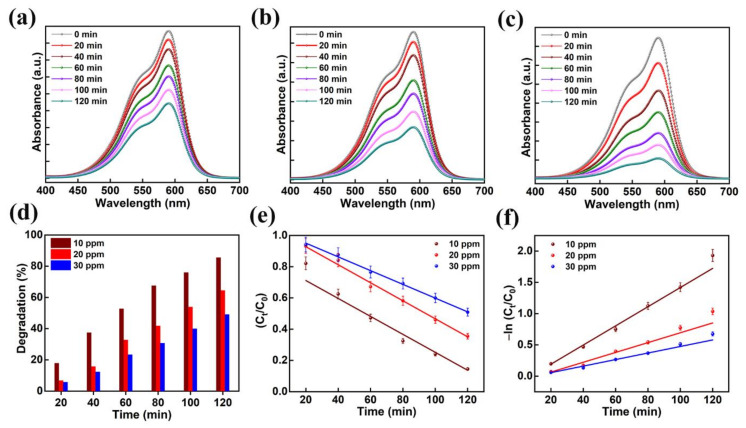
The absorption spectra for the presence of (**a**) 30, (**b**) 20, and (**c**) 10 ppm of CV dye amassed at regular intervals for 120 min of photocatalytic irradiation. (**d**) Histogram of dye removal efficiency, (**e**) kinetic plots, and (**f**) corresponding pseudo-first-order kinetic curves evaluated for photo catalytically degraded CV dye in the presence of AMOF nanosheets at numerous times of irradiation.

Photocatalytic activity was performed on several organic contaminants to ascertain the mechanism of dye degradation of the AMOF. The mechanism of photocatalytic activity was optimized with the help of the CV dye. The absorption spectra for various amounts of CV recorded at different times of the reaction are shown in Figure 7a–c. Primarily, optimization was achieved on CV dye for different amounts of 10, 20, and 30 ppm while maintaining the concentration of AMOF at approximately 50 mg. A strong absorption peak was noticed at 590 nm which belongs to the CV dye. There is no sign of another absorption peak further proving the lack of formation of dye-complexes. The absorption spectra scanned at various intervals of time for the photocatalytic removal of 30 ppm CV is demonstrated in Figure 7a. The intensity of the absorption peak is inversely proportional to the reaction time of the photocatalytic activity. The intensity of the absorption peak (i.e., λ_max_) gradually diminishes without any deviation in the position of the peak. This may further confirm that the dye has not produced any other complexes but decomposed photocatalytically, the extent depending on the irradiation times. Furthermore, the UV-visible absorption plots for 20 and 10 ppm CV dye are illustrated in Figure 7b,c, respectively. To evaluate the photocatalytic activity of AMOF for the removal of various amounts of CV dye, the percentage of dye removal was measured using Equation (1). Figure 7d displays the removal of the CV dye histogram at various irradiation times calculated from the UV-visible absorption curve. The increase in the photocatalytic irradiation time enables the AMOF gradually to achieve improvement in the percentage of dye degradation, while the maximum numbers are noticed after 120 min of irradiation regardless of CV dye concentration. The dye removal percentages of 49.1, 64.5, and 85.5% were attained for 30, 20, and 10 ppm CV after 120 min of irradiation with AMOF. Consequently, the excellent photocatalytic activity with 85.5% degradation was achieved for 10 ppm CV dye after 120 min of photocatalytic irradiation.

Additionally, the kinetic plots of CV dye pollutant removal by AMOF were investigated in detail. Depending on the photocatalytic duration the linear variation in ln (C_t_/C_0_) follows the kinetics of a pseudo-first-order reaction. The constant (k) value of the pseudo-first-order reaction rate was estimated using the following equation [46]:

ln (C_t_/C_0_) = −kt (3)

where, k is the constant of reaction rate, t is the duration of photocatalytic reaction, C_0_ and C_t_ are the absorbances at the initial concentration of dye (i.e., time t = 0) and the concentration of dye after light exposure (i.e., t = t), respectively.

Figure 7e,f indicates the plot of −ln (C_t_/C_0_) and C_t_/C_0_ with respect to irradiation time for the photocatalytic decomposition of CV dye. The reaction rate constant (k) values of 0.0153, 0.0078, and 0.0052 min^−1^. were estimated for the decomposition of 10, 20, and 30 ppm CV. The CV dye with the concentration of 10 ppm shows a higher rate constant value compared to the 20 and 30 ppm. samples The dye solution saturation on the surface of the catalyst leads to a reduction in active reaction sites, which could be the explanation. Likewise, diminished light penetration affects the dye degradation percentage. Further, we carried out photocatalytic decomposition analysis on the various organic dye pollutants with optimized 10 ppm concentration according to these investigations.

**Figure 8:**
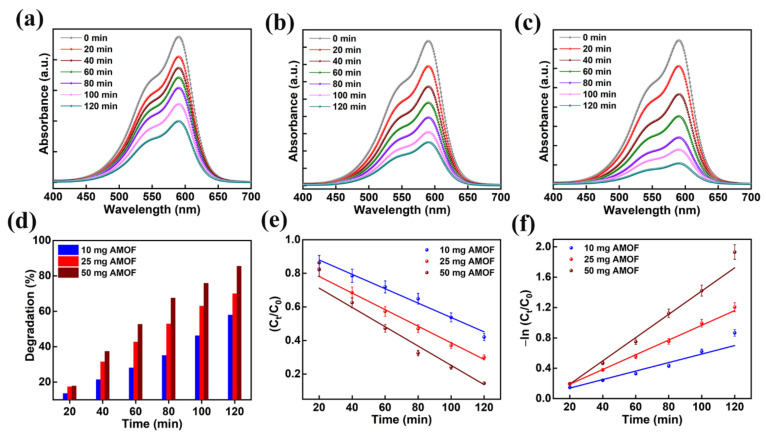
The absorption spectra in the presence of (**a**) 10, (**b**) 25, and (**c**) 50 mg of AMOF nanosheets of CV dye amassed at regular intervals for 120 min of photocatalytic irradiation. (**d**) Histogram of dye removal efficiency, (**e**) kinetic plots and (**f**) corresponding pseudo-first-order kinetic curves evaluated for photo catalytically degraded CV dye in the presence of AMOF nanosheets at numerous times of irradiation.

Moreover, the optimization of catalyst dosage is vital in validating the effective photon absorption to minimize wastage of catalyst for the decomposition of organic contaminants. Primarily, the dosage of AMOF photocatalyst was optimized using the mechanism of CV dye pollutant decomposition. The absorption plots of 10, 25, and 50 mg of AMOF nanosheets gained for photocatalytic CV dye pollutant removal are shown in Figure 8a–c. The absorption peak observed at 590 nm for CV dye gradually diminishes for all the dosages of AMOF nanosheets over 120 min of light irradiation. Furthermore, the reduction in absorption peak intensity is enhanced with the increase in concentration of catalyst. A substantial drop in intensity of the absorption peak is obtained for a dosage of 50 mg of photocatalyst. Figure 8d reveals the photocatalytic degradation efficiency as a function of irradiation time for all the concentrations of photocatalyst. The dye removal percentages of CV dye pollutant estimated for 10, 25, and 50 mg of AMOF catalyst were 57.9, 70, 85.5%, respectively. The superior CV dye removal percentage achieved for 50 mg of AMOF photocatalyst was accredited to the abundant electron and hole pair availability in the performance of photocatalytic action. In addition, the pseudo first order kinetic model was utilized to demonstrate the performance of photocatalytic activity. The plot of −ln (C_t_/C_0_) and C_t_/C_0_ with respect to the irradiation time shown in Figure 8e,f indicates the CV dye degradation. The values of pseudo first order rate constants (k) for AMOF of 10, 25, and 50 mg were estimated to be 0.005, 0.009, 0.015 min^−1^, respectively. These forecasted outcomes demonstrated that catalyst with 50 mg of concentration displays a very good performance for CV dye pollutant degradation. Therefore, 50 mg of AMOF nanosheets was utilized further to investigate the photocatalytic dye degradation of MB and MO.

**Figure 9:**
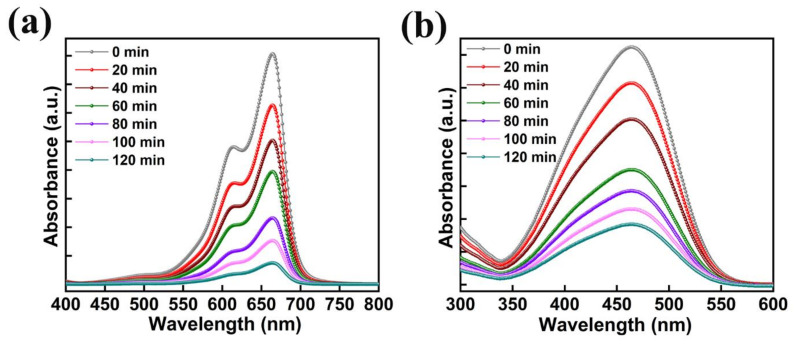
The absorption spectra with respect to the different illumination times of (**a**) MB, and (**b**) MO dyes collected at regular intervals for 120 min of photocatalytic irradiation in the presence of 50 mg of AMOF nanosheets.

The absorption plots of MB and MO with the usage of 50 mg of AMOF nanosheets gained at various time intervals of light irradiation are illustrated in Figure 9a,b. The peaks observed at 664 and 463 nm are the MB and MO dye pollutants absorption peaks. Throughout the study of photocatalytic activity, after the irradiation of light no extra peaks were noticed, thereby avoiding development of AMOF as well as dye derivatives. The absorption peak intensity of MB and MO dyes decreases with respect to the irradiation time which favors the improvement in the performance of their photocatalytic activity. Depending on the respective absorption peak, the photocatalytic decomposition percentage was estimated for MB and MO dyes. A negligible amount of MB dye removal was detected in the absence of AMOF photocatalyst (Figure S1) which corroborates the AMOF nanosheets importance in the dye degradation of MB. Therefore, the exceptional ability of pollutant absorption and photosensitizing effect of the AMOF nanosheets supported the utilization of an extensive amount of visible light to enhance the performance of the photocatalysis.

**Figure 10:**
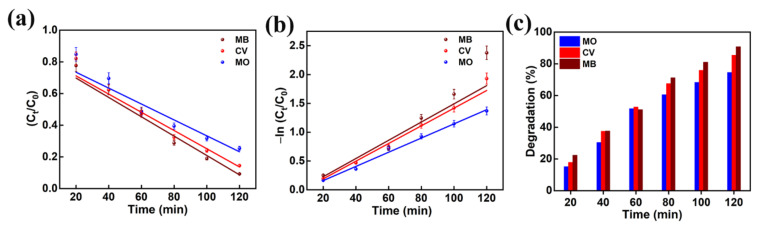
(**a**) Kinetic curves, (**b**) corresponding pseudo-first-order kinetic plots, and (**c**) histogram of dye removal efficiency evaluated for photo catalytically removed MO, CV, and MB dyes in the presence of 50 mg of AMOF nanosheets at numerous times of irradiation.

The performance of the photocatalytic decomposition was defined by the pseudo-first-order reaction kinetic plots. The plots of −ln (C_t_/C_0_) and C_t_/C_0_ as a function of irradiation time are presented in Figure 10a,b for the MO, CV, and MB dyes degradation predicted from Figure 9. The dye degradation mechanism using photocatalysis fitted well with pseudo first order kinetics. The pseudo first order rate constants (k) for MO, CV, and MB dyes were predicted to be 0.0123, 0.0153, and 0.0158 min^−1^, respectively. This reveals that the AMOF nanosheets presented a high rate constant in the case of MB dye and a low rate constant in the case of MO dye. Thus, it confirms that the AMOF nanosheets demonstrate a remarkable photocatalytic activity for MO, CV, and MB dye degradation. Figure 10c shows the photocatalytic dye degradation percentage histogram as a function of irradiation time estimated using the maximum absorption peaks noticed for MO (Figure 9b), CV (Figure 7c), and MB (Figure 9a) at 463, 590, and 664 nm. The dye removal percentages evaluated for MO, CV, and MB dye pollutants are 74.5, 85.5, 90.7%, respectively. This proves that the AMOF nanosheets display an enhanced photocatalytic performance for MB dye contaminant. To illustrate the benefits of current AMOF catalyst, the obtained outcomes in the MO, CV, and MB dye pollutants degradation were compared with already published reports provided in the Supplementary Information (Table S1).

**Figure 11:**
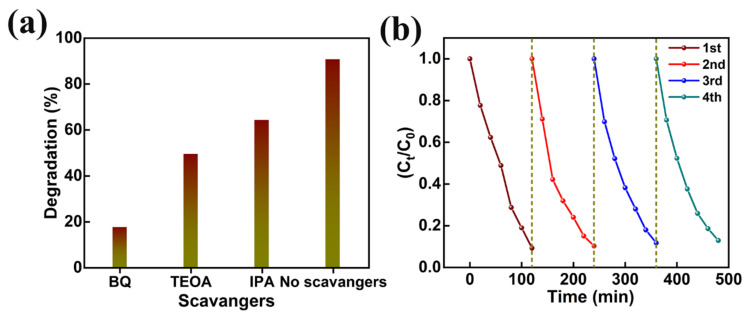
(**a**) Effect of different scavengers, and (**b**) cyclic stability study of AMOF nanosheets on photocatalytic removal of MB dye.

In addition, the effect of photogenerated holes (h^+^), hydroxyl radicals (OH^−^), superoxide radicals (O_2_^−^) in the photocatalytic decomposition of organic pollutants was investigated by various scavenger catching techniques using triethanolamine (TEOA), isopropanol (IPA), and benzoquinone (BQ), respectively. The MB degradation in the presence of TEOA, IPA, and BQ (Figure 11a) revealed greater quenching in MB degradation in the BQ presence. The strictly reduced MB dye degradation (i.e., 17.4%) in the presence of BQ supports the importance of superoxide radicals (O_2_^−^) in photocatalytic decomposition activity. Furthermore, the reasonable drop in dye degradation (i.e., 49.3%), detected in the presence of TEOA, corroborates the partial involvement of photogenerated holes (h^+^). Thus, MB dye removal is dominated by superoxide radicals (O_2_^−^) and photogenerated holes (h^+^). Moreover, the reusability of AMOF nanosheets was explored for four repeated cycles of MB dye decomposition (Figure 11b). The ~3.7% loss (i.e., from 90.7% to 87.0%) documented in the photocatalytic decomposition of MB dye pollutant can be attributed to the catalyst loss during the isolation approaches such as washing, centrifugation, and desiccating, etc. Moreover, a reusable study of AMOF nanosheets was investigated using XRD, FESEM, and FTIR spectra. The XRD and FTIR studies before and after photocatalytic activity of AMOF nanosheets (Figure S2a,b) were identical, and validate the exceptional stability of the photocatalytic activity. FESEM images of AMOF nanosheets after the photocatalytic reusability study are depicted in Figure S2c,d. The FESEM images of AMOF featured in Figure S2c,d demonstrate that the agglomeration of nanosheets take place without changes in surface morphology. Electrochemical impedance spectroscopy (EIS) was further utilized to understand the charge separation and charge carrier recombination; the corresponding Nyquist plot of EIS spectra for AMOF nanosheets is presented as shown in Figure S3. The impedance of the AMOF nanosheets can be determined according to the radius of semi-circular arc on the Nyquist plot. It was found that the smaller the radius, the lower the impedance and the higher the efficiency of photogenerated carrier separation. As is well known, higher electron-hole separation efficiency leads to higher photocatalytic activity, meaning that AMOF nanosheets show superior photocatalytic activity performance.

**Figure 12:**
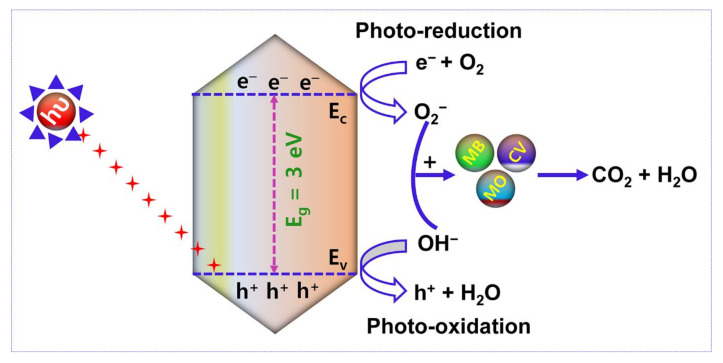
Schematic illustration of the photocatalytic performance of AMOF nanosheets in the removal of organic dye pollutants.

Figure 12 reveals the pictorial illustration of the mechanism of photocatalytic activity. After Xenon lamp irradiation, the electron (e^−^)-hole (h^+^) pair is generated and utilized to control the performance of the photocatalytic activity. Accordingly, the photocatalytic decomposition mechanism is based on (i) electron and hole arrest and recombination and (ii) the captured electron (e^−^)-hole (h^+^) pair recombination. The enhanced duration of e^−^-h^+^ pair recombination and the greater electron transfer rate boundary boost the organic pollutants degradation using photocatalytic activity. In this research work, the removal of organic dye pollutants by photocatalytic mechanism is defined as follows:

AMOF + hυ → AMOF (e^−^ + h^+^) (4)

AMOF (e^−^) + O_2_ → AMOF (*O_2_^−^) (5)

Dye + *O_2_^−^ → Dye_ox_ (intermediates) → CO_2_ + H_2_O (6)

AMOF (h^+^) + H_2_O → AMOF (OH^−^) + h^+^ (7)

Dye + OH^−^ → Dye_ox_ (intermediates) → CO_2_ + H_2_O (8)

Under the irradiation of light with reasonable energy of photons larger than or equivalent to the AMOF nanosheets bandgap, electrons (e^−^) travel to the conduction band (C.B) from the valence band (V.B.), leaving holes in the V.B., which are initiated and then reconnect on the AMOF nanosheets (Equation (4)). Further, the electrons are captured by AMOF and restrict the electron (e^−^)-hole (h^+^) recombination which enables the O_2_^−^ radicals to degrade different dye pollutants (Equations (5) and (6)). In almost all situations, holes will react with H_2_O to generate OH^−^ radicals (Equation (7)) [46]. It is well known that OH^−^ radicals are strong oxidizing agents capable of decomposing almost all organic contaminants. The OH^−^ radicals can be used to oxidize the dye pollutants (Equation (8)) which are thereby transformed to minor organic fragments, and these fragments can ultimately be mineralized to H_2_O and CO_2_. Thus, the larger surface area and morphology of the AMOF nanosheet may have a significant influence on low cost and feasible photocatalytic activity in the decomposition of various dye pollutants.

## 4. Conclusions

In summary, AMOF nanosheets were designed by the solvothermal route and utilized as a photocatalyst to degrade various organic pollutants. The morphology and structural studies confirmed the AMOF nanosheets formation. From the UV-visible spectroscopy a bandgap of 3.0 eV was determined. The AMOF nanosheets displayed remarkable performance of photocatalytic activity for the removal of MO, CV, and MB dyes under the illumination of a Xenon lamp. Dye removal percentages of 74.5, 85.5, and 90.7% for MO, CV, and MB dyes were obtained for the optimized photocatalytic activity. The AMOF nanosheets revealed the highest percentage of dye removal in the case of MB compared to the other dyes. Furthermore, stable photocatalytic activity and recyclability of AMOF nanosheets were determined using XRD and FTIR spectra and without the creation of other organic pollutants and structural changes. The substantial influence of superoxide radicals and photogenerated holes on the photocatalytic removal of dye pollutants was studied using radical scavenger trapping experiments. Overall, our study on AMOF nanosheets may open up a possible pathway to explore MOF based materials as a beneficial photocatalyst for the development of a low cost and environmentally friendly route for the removal of organic pollutants.

## Data Availability

Data will be available on request.

## Supplementary Materials

The following supporting information can be downloaded at: https://www.mdpi.com/article/10.3390/nano13020336/s1, Figure S1. (a) The absorption spectra, and (b) dye removal efficiency histogram of MB dye amassed at regular intervals for 120 min photocatalytic illumination in the absence of photocatalyst; Figure S2. (a) XRD spectra, and (b) FTIR spectra attained from the AMOF nanosheets before and after four repeated photocatalytic decomposition cycles of MB dye. (c,d) FESEM images obtained from the AMOF nanosheets after four repeated photocatalytic decomposition cycles of MB dye; Figure S3. Electrochemical impedance spectroscopy plot of AMOF nanosheets; Table S1. Comparison of photocatalytic activities of AMOF nanosheets with some reported literatures available on photocatalysts in the decomposition of various organic dye pollutants [Methyl Orange (MO), Crystal Violet (CV), and Methylene Blue (MB)].
