# Supplementary material for: Development of MOF Based Recyclable Photocatalyst for the Removal of Different Organic Dye Pollutants
Source: Nanomaterials (Basel). 2023 Jan 13;13(2):336. doi: 10.3390/nano13020336 (PMC9861624; doi:10.3390/nano13020336)
Supplement: Supplementary file 1 [file nanomaterials-13-00336-s001.zip › nanomaterials-2157822-supplementary.pdf]

Supplementary Information

Development of MOF based recyclable photocatalyst for the removal of different organic dye pollutants

Narasimharao Kitchamsetti [a,b,], Chidurala Shilpa Chakra [b], Ana L. F. de Barros [c], Daewon Kim [a,]**

[a] *Department of Electronic Engineering, Institute for Wearable Convergence Electronics, Kyung Hee University, 1732 Deogyeong-daero, Giheung-gu, Yongin 17104, Republic of Korea*

[b] *Center for Nano Science and Technology, Institute of Science and Technology, JNTU Hyderabad, Hyderabad 500090, India*

[c] *Laboratory of Experimental and Applied Physics, Centro Federal de Educação Tecnológica Celso Suckow da Fonseca, Av. Maracanã Campus 229, Rio de Janeiro 20271-110, Brazil*

* Corresponding authors

Email address: kitchamsetti.rao@khu.ac.kr (N. Kitchamsetti),

daewon@khu.ac.kr (Daewon Kim)

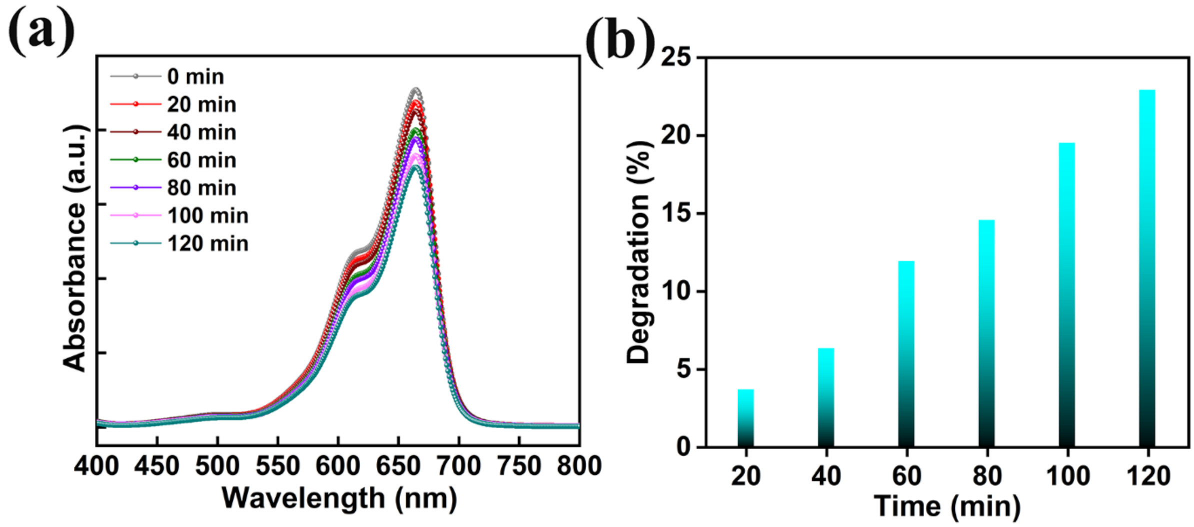

Figure S1. (a) The absorption spectra, and (b) dye removal efficiency histogram of MB dye amassed at regular intervals for 120 min photocatalytic illumination in the absence of photocatalyst.

Table S1. Comparison of photocatalytic activities of AMOF nanosheets with some reported literatures available on photocatalysts in the decomposition of various organic dye pollutants [Methyl Orange (MO), Crystal Violet (CV), and Methylene Blue (MB)]

Sample	Dye	Light source	Photodegradation		Ref.
			Time (min.)	Amount (%)	
Zn-MOF	RhB	UV light	60	55.1	[1]
MIL-88A(Fe)	RhB	Visible light	80	45	[2]
MIL-88A(Fe)/GO	RhB	Visible light	80	94	[2]
Silver vanadate/BiFe-MOF	RhB	Visible light	120	99.4	[3]
Cu doped MOF-235	MB	Visible light	8 h	90	[4]
ZnO@MIL-101(Fe)	RhB	Visible light	300	97.1	[5]
Mn-MOF	MB	UV light	70	90.8	[6]
MIL53Fe@MIL53Sr	RhB	UV light	60	75	[7]
Ni-MOF	CV	Xe lamp	30	93	[8]
Cu-MOF	RhB	Visible light	60	99	[9]
Fe/Ce-MOF	MO	Visible light	30	93	[10]
Bi-MOF	RhB	Visible light	180	99.1	[11]
Co-MOF	MB	Visible light	80	95	[12]
Ni/Co-MOF	MB	Xe lamp	120	80.6	[13]
(TiO_2@Cd-MOF)@ZnPp	MO	Visible light	60	94.1	[14]
CdSe@Fe-BDC	RhB	Visible light	240	99.8	[15]
N-TiO@MIL-100(Fe)	RhB	Visible light	180	93.5	[16]
AMOF nanosheets	MO	Xe lamp	120	74.5	This work
	CV			85.5	
	MB			90.7	

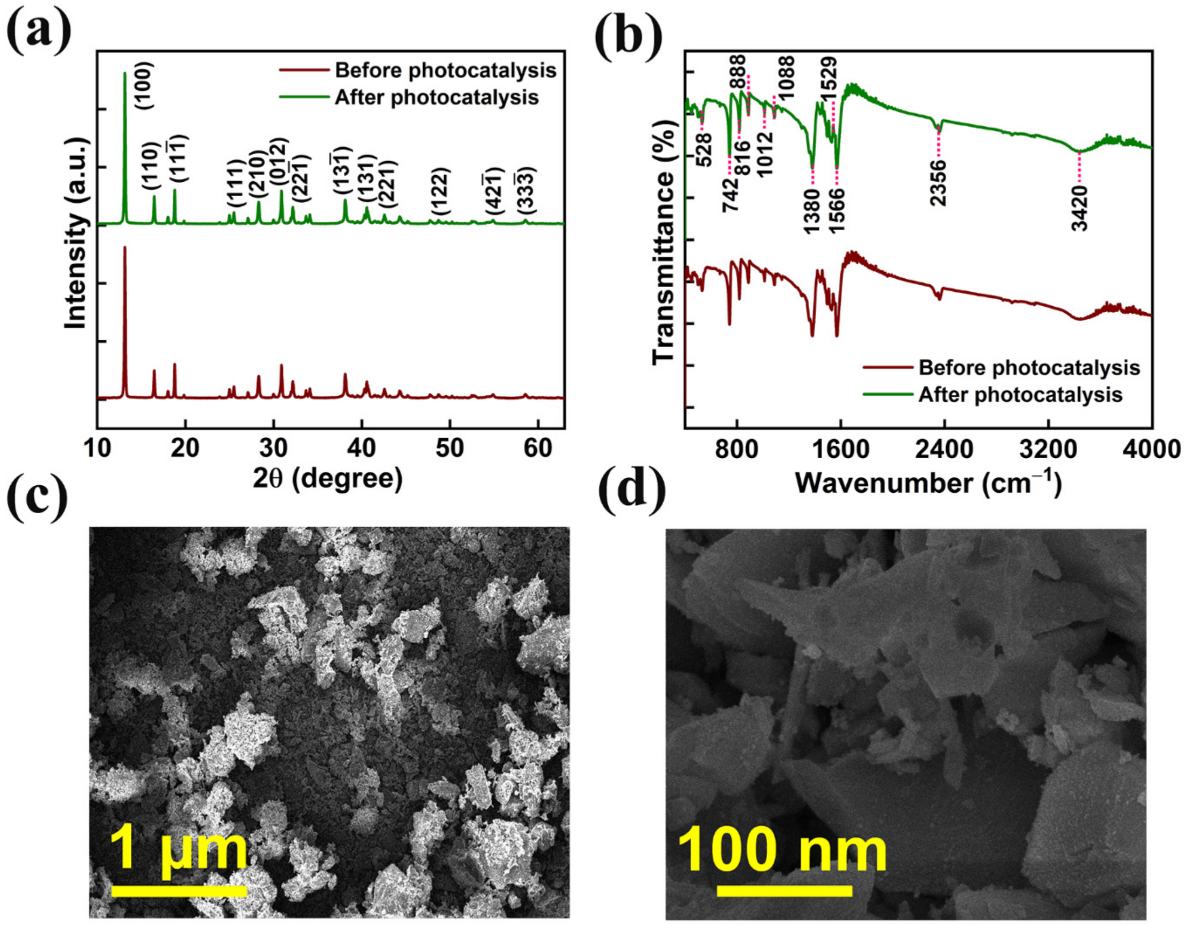

Figure S2. (a) XRD spectra, and (b) FTIR spectra attained from the AMOF nanosheets before and after four repeated photocatalytic decomposition cycles of MB dye. (c, and d) FESEM images obtained from the AMOF nanosheets after four repeated photocatalytic decomposition cycles of MB dye.

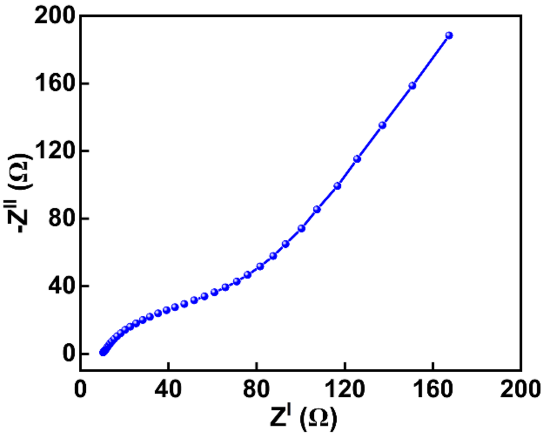

Figure S3. Electrochemical impedance spectroscopy plot of AMOF nanosheets.

References

[1] W.P. Wu, Application of a water stable Zinc (II) glutamate metal organic framework for photocatalytic degradation of organic dyes, Bull. Chem. Soc. Ethiop. 33 (2019) 43-50, https://dx.doi.org/10.4314/bcse.v33i1.4.

[2] N. Liu, W.Y. Huang, X.D. Zhang, L. Tang, L. Wang, Y.X. Wang, M.H. Wu, Ultrathin graphene oxide encapsulated in uniform MIL-88A(Fe) for enhanced visible light-driven photodegradation of RhB, Appl. Catal.B-Environ. 221 (2018) 119-128, https://doi.org/10.1016/j.apcatb.2017.09.020.

[3] W.Y. Lou, L. Wang, S.H. Dong, Z.Z. Cao, J.M. Sun, Y.F. Zhang, A facility synthesis of bismuth-iron bimetal MOF composite silver vanadate applied to visible light photocatalysis, Opt. Mater. 126 (2022) 112168, https://doi.org/10.1016/j.optmat.2022.112168.

[4] N.T. Tran, D.K. Kim, K.S. Yoo, J.S. Kim, Synthesis of Cu-doped MOF-235 for the Degradation of Methylene Blue under Visible Light Irradiation, Bull. Korean Chem. Soc. 40 (2019) 112-117, https://doi.org/10.1002/bkcs.11650.

[5] E. Amdeha, R.S. Mohamed, A green synthesized recyclable ZnO/MIL-101 (Fe) for Rhodamine B dye removal via adsorption and photo-degradation under UV and visible light irradiation, Environ. Technol. 42 (2021) 842-859, https://doi.org/10.1080/09593330.2019.1647290.

[6] X.D. Fang, L.B. Yang, A.N. Dou, Y.E. Liu, J. Yao, Q.Q. Xu, A.X. Zhu, Synthesis, crystal structure and photocatalytic properties of a Mn (II) metal-organic framework based on a thiophene-functionalized dicarboxylate ligand, Inorg. Chem. Commun. 96 (2018) 124-127, https://doi.org/10.1016/j.inoche.2018.08.017.

[7] M. Humayun, M.M. He, W.B. Feng, C.Y. Jin, Z. Yao, Y.C. Wang, W.B. Pi, S. Ali, A. Khan, M. Wang, Z.P. Zheng, Q.Y. Fu, H. Xia, W. Luo, Enhanced photocatalytic

performance of novel MIL53Sr metal-organic framework (MOF) for RhB dye degradation and H_2 evolution by coupling MIL53Fe, Solar Energy 215 (2021) 121-130, https://doi.org/10.1016/j.solener.2020.12.025.

[8] K.C. Devarayapalli, S.V.P. Vattikuti, T.V.M. Sreekanth, K.S. Yoo, P.C. Nagajyothi, J.S. Shim, Facile synthesis of Ni-MOF using microwave irradiation method and application in the photocatalytic degradation, Mater. Res. Exp. 6 (2019) 1150h3, https://doi.org/10.1088/2053-1591/ab5261.

[9] M.S. Samuel, K.V. Savunthari, S. Ethiraj, Synthesis of a copper (II) metal-organic framework for photocatalytic degradation of rhodamine B dye in water, Environ. Sci. Pollut. Res. 28 (2021) 40835-40843, https://doi.org/10.1007/s11356-021-13571-9.

[10] T. Tang, X.H. Jin, X.M. Tao, L. Huang, S. Shang, Low-crystalline Ce-based bimetallic MOFs synthesized via DBD plasma for excellent visible photocatalytic performance, J. Alloys Compd. 895 (2022) 162452, https://doi.org/10.1016/j.jallcom.2021.162452.

[11] V.H. Nguyen, A.L.H. Pham, V.H. Nguyen, T.Y. Lee, T.D. Nguyen, Facile synthesis of bismuth(III) based metal-organic framework with difference ligands using microwave irradiation method, Chem. Eng. Res. Des. 177 (2022) 321-330, https://doi.org/10.1016/j.cherd.2021.10.043.

[12] L. Liu, Q. Chen, Z.B. Han, A Co(II)-based non-interpenetration semiconductive metal-organic framework for photocatalytic degradation of organic dye contaminant, Inorg. Chem. Commun. 138 (2022) 109224, https://doi.org/10.1016/j.inoche.2022.109224.

[13] C.C. Shan, X. Zhang, S.M. Ma, X.X. Xia, Y.X. Shi, J. Yang, Preparation and application of bimetallic mixed ligand MOF photocatalytic materials, Colloid Surf. A-Physicochem. Eng. Asp. 636 (2022) 128108, https://doi.org/10.1016/j.colsurfa.2021.128108.

[14] V. Ramasubbu, P.R. Kumar, T. Chellapandi, G. Madhumitha, E.M. Mothi, X.S. Shajan, Zn (II) porphyrin sensitized (TiO_2@Cd-MOF) nanocomposite aerogel as novel

photocatalyst for the effective degradation of methyl orange (MO) dye, Opt. Mater. 132 (2022) 112558, https://doi.org/10.1016/j.optmat.2022.112558.

[15] Y. Zhang, G. Li, Q.Y. Guo, CdSe QDs@Fe-based metal organic framework composites for improved photocatalytic RhB degradation under visible light, Micropor. Mesopor. Mater. 324 (2021) 111291, https://doi.org/10.1016/j.micromeso.2021.111291.

[16] J. Huang, H.Y. Song, C.X. Chen, Y. Yang, N.D. Xu, X.Z. Ji, C.Y. Li, J.A. You, Facile synthesis of N-doped TiO_2 nanoparticles caged in MIL-100(Fe) for photocatalytic degradation of organic dyes under visible light irradiation, J. Environ. Chem. Eng. 5 (2017) 2579-2585, https://doi.org/10.1016/j.jece.2017.05.012.
